# Perspective of healthy asymptomatic patients requesting general blood tests from their physicians: a qualitative study

**DOI:** 10.1186/s12875-019-0940-9

**Published:** 2019-04-05

**Authors:** Michal Shaked, Inbar Levkovich, Tamar Adar, Alma Peri, Nir Liviatan

**Affiliations:** 1 Department of Family Medicine, The Division of Family Medicine, The Ruth & Bruce Rappaport Faculty of Medicine, Technion-Israel Institute of Technology, 6 Hashachaf St, Bat-Galim, 35013 Haifa, Israel; 2 Faculty of Graduate Studies, Oranim Academic College of Education, Kiryat Tiv’on, Israel; 3 Department of Family Medicine, The Ruth & Bruce Rappaport Faculty of Medicine, Technion-Israel Institute of Technology, Clalit Health Services, Haifa, Western Galilee District Israel

**Keywords:** Blood test, Asymptomatic, Primary care, Family physician, Request, Reason

## Abstract

**Background:**

Routine blood tests for young, healthy, asymptomatic patients have no proven value in early detection of diseases. Indeed, such tests have occasionally been found to be harmful. Although general blood tests are not recommended by evidence-based guidelines, patients frequently request referrals for these tests. A number of studies have examined the factors influencing doctors to prescribe such tests, yet little is known about patients’ perspectives on this topic. The present study evaluated the knowledge, attitudes and beliefs of young, healthy asymptomatic patients requesting general blood tests from their family physician.

**Method:**

Qualitative interviews with 15 healthy, asymptomatic patients aged 22–50 who requested general blood tests from their family physicians. We conducted in-depth semi-structured interviews within two weeks of their request.

**Results:**

Three main themes emerged from the interviews: 1) Patients’ sense of personal responsibility and their belief that periodic blood tests are beneficial as an integral part of their health maintenance. 2) Patients’ need to receive external, objective and reliable validation about what is happening inside their bodies. 3) An acquaintance’s serious illness as a prompt to perform general blood tests in the belief that such tests can reveal latent conditions.

**Conclusion:**

The study revealed a substantial gap between patients’ attitudes and beliefs about general blood tests and current evidence-based guidelines. Implications for research and practice are discussed.

## Background

Routine blood tests for young, healthy patients are not recommended by evidence-based guidelines, except for a lipid profile once every five years for men over 35 and women over 40 [1–3]. Large prospective studies have failed to demonstrate the efficacy of routine blood tests for the general population for early detection of diseases [4–9], and such tests have even occasionally been found to be harmful [10, 11].

The total accumulated cost of these blood tests is high and false positive results are frequent. Such results may cause anxiety and lead to unnecessary referrals for additional testing and investigation [12, 13]. A study conducted with over 180,000 patients showed that performing general blood tests increased diagnoses and treatments without significant effects on mortality [14].

While blood tests may reveal conditions such as anemia, renal or liver dysfunction, and diabetes mellitus, blood tests are in fact mostly effective when performed on the basis of suspicious findings arising from a thorough history and physical examination. Despite the fact that many abnormal results may be false positives [15], they still often require further investigation, leading to additional medical expenses and patient anxiety, as well as potential physical harm due to exposure to radiation, contrast material and invasive procedures. Unnecessary tests may also lead to overdiagnosis and prompt unnecessary treatment [16], the cost of which is estimated at more than 200 billion dollars every year in the United States [17]. Furthermore, discussing test results with patients is time consuming. Time spent discussing the results of blood tests that were unnecessary to begin with might be better utilized discussing evidence-based recommended tests and lifestyle modifications as a means of disease prevention.

In the past two decades, blood testing in the ambulatory setting has markedly increased [18–20]. There are numerous reasons for this increase pertaining to doctors, patients, society, technological advances and the medical system. Shifts in trends and financial objectives of various interest groups may also play a role [21, 22].

A number of studies have been conducted to examine the reasons for performing unnecessary blood tests. Factors influencing doctors to prescribe tests were examined in a number of studies. Little et al. examined the influence of patient pressure on doctors to provide prescriptions and referrals [23]. The study showed that in 46% of cases, doctors felt their referral was unwarranted but nevertheless fulfilled the patient’s request. Perceived patient pressure was cited as the most influential factor. Other studies attempted to reveal an association between physician characteristics and blood test referrals. The physician’s personality and difficulty dealing with uncertainty were substantial factors. Referrals were also associated with gender, age, years of experience and workload [24–32]. Another study demonstrated that patient expectations and fear of legal action were additional influential factors [33].

The reasons that asymptomatic patients request blood tests have been studied less frequently. Two quantitative studies that examined the reasons why asymptomatic patients request check-ups found there may be covert reasons or hidden agendas behind these requests, among them fear of illnesses such as cancer, AIDS and heart disease; a history of psychosocial problems; and experiencing worrisome symptoms or symptoms patients believed could be easily diagnosed by blood tests [34, 35]. One qualitative study was conducted in Holland in 2006 among patients who visited primary care clinics requesting blood tests, but was not restricted to asymptomatic patients. The study found that patients perceived favorable test results as proof of good health and regarded blood tests as a useful screening instrument for serious disorders [36]. Patients overestimated the ability of blood tests to confirm health and diagnose covert illness. Moreover, patients expected diagnostic certainty without error [36]. The discrepancy between patients’ beliefs about various tests and the actual proven efficacy of these tests has been demonstrated in other studies as well [37–39].

In Israel, patients’ reasons for requesting blood tests have not been studied to date.

Israel’s primary health care system is public and based on mandatory, government-subsidized health insurance. The cost of nearly all blood tests is covered by health insurance. A patient may request tests either directly during a doctor visit or via online or telephone request. Requests for most tests must be authorized by a physician. Considering the paucity of current research on the perspective of asymptomatic patients who request general blood tests from their family physician and the potential costs and risks of unnecessary tests, we conducted a qualitative study to improve our understanding of these patients’ perceptions and beliefs regarding blood tests.

## Method

The study used a qualitative-phenomenological approach. *Qualitative phenomenological research* seeks to identify phenomena through how they are perceived by the actors in a situation. It entails studying individuals’ lived experiences of a phenomenon and reducing these experiences to a description of their universal essence [40, 41]. This study explores how healthy, asymptomatic patients perceive their requests for general blood tests from their general practitioner.

### Sample and population

This qualitative study was supported by a grant from the Israel Medical Association. The sample consisted of 15 patients recruited from five primary care clinics in northern Israel. Based on the purposeful sample approach, we chose clinics that best represented Israel’s heterogeneous population in terms of age, gender, education and socioeconomic status, and that could best teach us about the studied phenomenon [42]. Inclusion criteria for the study were individuals aged 20–50, Hebrew speakers, otherwise healthy and asymptomatic who asked their family physician for a blood test referral.

### Research procedure

The study was approved by the Institutional Review Board of the Haifa and Western Galilee District of Clalit Health Services (the largest public health organization in Israel) (Approval No. 0221–16-COM2). The researchers recruited participants with the assistance of primary care physicians. Nine physicians from five primary care clinics in the Haifa and Western Galilee district participated in the study. Four of the clinics are urban and one is rural. The physicians were instructed to identify patients who came in for an appointment and requested general blood tests and who met the inclusion criteria, and to ask them whether they were willing to participate in the study. Patients who consented were referred to the researchers. This method of recruiting patients was chosen in order to focus on those patients who consider blood tests important enough to warrant a visit to their physician specifically for this purpose, as this is the target population.

Patients received a comprehensive explanation about the study and signed informed consent forms. They could withdraw from the study at any time and could refuse to answer any question. No incentives were offered. The interview was set within two weeks of referral. The interviews were conducted in Hebrew, recorded and translated into English. Each translation was verified by two native English speakers, one of whom is a certified translator. The interviews were conducted at the patient’s home or at the clinic, according to the participant’s preference, while ensuring medical confidentiality. The interviews lasted approximately one hour. Data collection commenced in May 2017 and was completed by June 2018, when theoretical saturation was reached (i.e., additional interviews yielded no new material for analysis).

### Research tools and instruments

The qualitative data in this study were gathered using semi-structured, in-depth interviews [42]. Three interviewers conducted the interviews. They were selected based on their previous experience and expertise. Two of the interviewers were family medicine practitioners, and one was a family medicine resident. Interviewers received training in qualitative interviewing. The interview was used as an instrument to help us learn about and examine the participants’ views and was conducted based on an interview guide (Table 1) that included significant key areas but was flexible enough to allow for a dialogue to develop between interviewer and interviewee and for meaningful self-expression [42]. The interview guide deliberately covered broad topics because the authors sought to promote open and uninhibited discussion in order to obtain authentic input from the patients.

**Table 1:** Interview questions posed to young, healthy and asymptomatic patients requesting general blood tests from their family physician

1	What kind of concerns, if any, do you have regarding your health? How often do you experience these concerns?
2	What do you know about early detection of diseases?
3	What were your reasons for requesting general blood tests from your family physician?
4	What specific blood tests were you interested in doing when requesting general blood tests from your doctor?
5	How often do you think a healthy patient your age should have blood tests?
6	Did anyone close to you recently have a severe illness? What effect did that have on your wish to have blood tests done?
7	Do you read/watch reports about health issues in the news/internet/TV? How did these affect your wish to perform general blood tests?
8	What kind of effect do you think the blood test results will have on your physical and mental health and lifestyle?
9	How will you feel if some of the blood test results are out of the normal range but your family physician explains that this has no significant meaning? Will you be interested in further investigations?
10	When requesting general blood tests from your family physician, how do you see his/her role? Would you like to hear his/her opinion regarding the necessity of the tests?
11	How did your family physician react to your request for general blood tests? How did you feel about his/her response? Do you feel his/her response affected your relationship with him/her? how?

### Data analysis

When the interviews were completed, they were transcribed verbatim and the data were analyzed thematically [43]. Data categories were coded in the following stages: 1) Open coding: Two researchers separately read each interview transcript line by line and jotted down notes to capture and identify initial units of meaning (categories) emerging from the data. Commonalities and differences across interviews were evaluated and themes were regrouped to represent major content areas that received considerable attention across participants. 2) The researchers then reviewed the larger themes, discussed disparities and sought agreement concerning theme content and interpretation of meaning. 3) Axial coding: In a second reading of the transcripts, the researchers gradually detected associations between the themes and sub-themes related to context and content. They compared all completed interviews so as to consolidate meaning and arrive at a theoretical construct. 4) Integration: The researchers identified the study’s central themes. The core themes that emerged from the data were reordered conceptually and placed back into context, making it possible to analyze and integrate large amounts of data and to generate abstractions and interpretations [43].

## Results

Fifteen patients participated in this study: ten males and five females. Eleven of the patients were between the ages of 20 and 35, and four were between the ages of 35 and 50. Most participants were urban clinic patients (Table 2). Twelve patients obtained the requested referral from their physician. Six of them had already completed the blood tests before the interview took place.

**Table 2:** Participant characteristics

Characteristics of 15 participating patients	*n* (%)
Gender	
Female	5 (33%)
Male	10 (66%)
Age	
20–35 years	11 (73%)
35–50 years	4 (27%)
Practice location	
Urban	13 (87%)
Rural	2 (13%)

Qualitative analysis of the interviews revealed three central themes: The first theme demonstrates a sense of responsibility on the part of young, healthy persons, together with a belief that they should have periodic blood tests as an integral part of their health maintenance. The second theme relates to the patients’ need to receive external and reliable validation about what is happening inside their bodies. The third theme deals with the effect of a serious illness experienced by a family member, friend or acquaintance on the patient’s desire to perform tests. In all three themes patients desire early detection of latent conditions, but the reasons differ between themes.

### Theme 1: “Blood tests are required once a year, like dental exams”: request for blood tests stems from a sense of responsibility

Many of the interviewees reported perceiving themselves as healthy. In order to maintain their health, they perform periodic blood tests. Most patients felt that once a year seemed like a reasonable period of time. Two patients compared performing regular blood tests to their regular bi-annual dental examination. Most of these patients requested “general” or “extensive” blood tests, but did not specify which particular tests they wished to perform.

*"I think every young person, whoever he may be and whatever his state of health, should have (blood) tests once a year, even if everything is all right. Like a dental checkup … I requested the most extensive tests, in order to get a picture of everything" (Patient 10).*

*"In a chat with some friends I asked: ‘How often do you think one should have a blood test?’ And they all said once a year … It's common knowledge … " (Patient 15).*

Several patients explained that even if they are not overly concerned about their health on a daily basis, periodic blood tests are the right and responsible thing to do for early detection of latent diseases. They shared the premise that blood tests can actually reveal all, or at least most, diseases, and that early diagnosis is always beneficial and has clear life-saving potential. Some considered not having blood tests to be neglectful.

*“It can save lives … it’s always preferable. There are times when it’s too late when you feel something in your body. So yes, it is always preferable to have all sorts of routine tests … ” (Patient 2).*

Most of the patients who expressed a sense of responsibility for early diagnosis through periodic blood tests and believed these tests were essential to their health stated that they expected their doctor to comply with their request. Many of the patients received an explanation from their physician about the blood tests being unnecessary but still obtained a referral. Most patients stated they were interested in hearing the doctor’s view but expected to obtain the referral even after the doctor explained why the requested tests are unwarranted. One patient who did not obtain a referral after a thorough explanation decided to leave her physician.

*“If the doctor had told me that blood tests are unnecessary? That wouldn’t satisfy me. Because I know for a fact that people who don’t do certain tests, even if they maintain a healthy lifestyle … it’s not enough … that’s why you have to know if there is something wrong … ” (Patient 3).*

*"I think what my doctor said is true. I understand what he said, it makes sense, but on the other hand, I feel that yes, once a year it [routine blood tests] needs to be done … " (Patient 14).*

A few patients reported that they adhere to a healthy lifestyle, including exercise, a healthy diet and stress-reduction techniques. They added routine blood tests to this list as part of the effort invested in their health. Other patients stated they are aware that even though they smoke, do not exercise and do not maintain a healthy diet, they at least try to have blood tests once a year in order to maintain their health.

*“I maintain my health with a proper diet, some exercise, positive thinking, and tests every now and then, to make sure nothing is wrong” (Patient 1).*

According to some of the interviewees, the assumption that blood tests are the appropriate method for early diagnosis and that early diagnosis is always recommended was further endorsed by the media. They claimed to have been exposed to health information recommending regular checkups in order to avoid delayed diagnosis of disease, which could result in dire outcomes, such as amputation or metastases.

*“I came in [to request blood tests] because I saw a report on diabetes on television. They said it could lead to limb amputation, etc. … I’m sure that report caused many people to go get tested … ” (Patient 9).*

A few patients mentioned the fact that there is no harm in having blood tests as their rationale for having them periodically. They explained that since there is no reason not to get tested and since blood tests cause no harm, it is definitely advisable to get tested.

*“I come in, do the tests once a year. No harm done. How much time does it take me? Ten minutes?”(Patient 13).*

### Theme 2: “Things go on inside your body, and often we don’t know what’s happening”: requesting blood tests for external validation of the body’s inner workings

Patients conveyed the feeling that one cannot trust what one feels: Even if one feels well, there is no way of knowing what is actually happening inside the body. Some patients described the body as an entity whose workings humans cannot understand. Thus, some sort of external monitoring is necessary to obtain reliable information about the body. These patients tend to attribute this capacity to blood tests.

*“We’re just a box of chemicals. What you put in, is what you get … I expect to see where I stand today on all measures, so I can fix and improve the situation … ” (Patient 10).*

*“Blood tests somehow give me peace of mind. They tell me: ‘Okay, everything is all right’. Because I don’t know what’s going on inside … the tests enable me to see or get an idea of something I can’t see, which is inside the body … ” (Patient 13).*

In particular, patients who follow a specific diet and feel the need to validate their efforts as worthwhile seek reliable external monitoring. They want to ensure they are not causing various deficiencies in their bodies. These patients tended to have more specific requests. In addition to general tests, most felt it was important to check for vitamins and iron.

*“I’ve been on a vegetarian diet for a year now. I want to know if something is deficient – iron, B12, I don’t know … I have to know everything is all right in order to continue with my healthy diet … ” (Patient 4).*

### Theme 3: “My father died of cancer. I’m afraid of getting sick”: requesting blood tests after exposure to a close person with a serious disease

A number of patients mentioned someone close to them who was diagnosed with a serious illness, such as cancer or heart disease. Some told about a family member who had a serious illness, usually cancer that had been diagnosed by chance, thanks to testing. This type of exposure brought about a sense of urgency to do periodic testing for early diagnosis of disease. These patients feared they were at greater risk because of their family history and therefore required testing. Moreover, in some cases, witnessing someone close to them who had been saved by tests reinforced their belief that routine tests save lives.

*“My grandmother died of cancer. Five years earlier she was tested and diagnosed with an early stage tumor, which they managed to suppress, giving her five more years [of life] … How can you achieve an early diagnosis? Mainly by blood tests … ” (Patient 13).*

Other patients described totally healthy young people around them who suddenly got sick or died. They talked about their distress over these cases and concluded that the only way to prevent the same thing from happening to them was by having routine blood tests. These patients expressed their personal fears and concerns. Their exposure to illness and the loss of someone close forced them to confront the fragility of life and made them suspect that a disease might be incubating within them. To alleviate their anxiety, they asked their doctor for blood tests.

*“One guy at work, a really unbelievable story … broke his arm one day at work. He was generally healthy. Then, when they x-rayed his arm, they discovered he had cancer that had spread all over his body … and within three months he died … ” (Patient 8).*

## Discussion

The objective of the present study was to examine the perceptions of healthy asymptomatic patients who request general blood tests from their family physicians. The results demonstrate three main incentives for requesting blood tests: 1) the belief that periodic blood tests are important and that requesting them is the responsible thing to do as an integral part of health maintenance; 2) the need for external validation of the body’s inner workings; and 3) anxiety due to exposure to illness in a close acquaintance. Our study shows that many patients believe blood tests provide information about their health status. The accepted medical approach—that blood tests are unnecessary when a patient feels well—does not correspond with patients’ strong need to know what is going on inside their bodies.

### Findings and comparison to the literature

The results of the current study reflect a substantial gap between patients’ beliefs about general blood tests and current accepted medical standards. On one side of this gap are the family physicians. Medical evidence-based knowledge shows that general blood tests are not useful in early detection of disease [4, 5]. Early diagnosis is not possible for every disease, nor will it affect the outcome of every disease. Doctors are aware that blood test results are statistical and have standard deviations. Deviation from the norm explains a large part of the results that fall outside the normal range. Doctors also understand that every test may entail undesirable consequences, including waste of time and money, patient anxiety, and further testing that may entail unnecessary radiation, contrast material or invasive procedures [3–5]. On the other side of the gap are the patients, who, according to the results of this study, wish to maintain their health and believe that periodic blood tests have the power to validate their health or diagnose early stages of disease before symptoms appear. They believe that early diagnosis is important, feasible and life-saving and that blood tests can achieve this. These results are in line with previous studies [36–38] and add the aspect of getting periodic blood tests as being responsible for one’s own health.

A few studies have examined physician characteristics and perspectives affecting test referrals [24–34]. Yet our review of the literature revealed a paucity of information regarding patients’ reasons for requesting tests in general, and blood tests in particular. Two qualitative studies examined the reasons that asymptomatic patients request general check-ups (though not specifically blood tests). These studies found that in many cases these patients had covert reasons, such as psychosocial reasons or health concerns [34, 35]. This is in line with the third theme demonstrated in our results, namely anxiety due to illness of a close acquaintance, and exemplifies the need for doctors to be aware of and sensitive to this possibility when faced with a patient who requests tests. We found only one previous qualitative study that focused on the specific question of why patients want to have their blood tested. In this study, 22 patients from five clinics were interviewed, resembling our sample. As opposed to our study, however, the inclusion criteria did not specify asymptomatic patients, and only four patients requested tests for screening purposes [36]. In line with our findings, this study found that patients overestimated the ability of blood tests to confirm good health and to detect serious conditions at an early stage. The study’s results also showed that these beliefs were intensified by the social environment and by the media. The consistency of findings after a period of over a decade and in a different geographical and cultural setting strengthens the validity of both studies. To the best of our knowledge, no previous studies described the sense of responsibility described by patients in our study to have their blood tested periodically as part of their effort to maintain their health. In reviewing the literature we also did not find patients’ need to monitor their inner body functions as a motive for requesting blood tests. We assume that these two themes have emerged over the past few years alongside cultural, social and technological trends.

The current study shows that many patients perceive periodic blood tests to be part of a pro-active and responsible approach to maintaining their general health. The physician’s view that blood tests should be performed as ancillary tests in a workup of symptoms is contrary to their outlook and seems neglectful to them. In the past few years, a gradual change has evolved in the physician-patient relationship such that it has become less paternalistic and more patient-centered [43]. Many patients visit the doctor with a specific agenda of obtaining a referral for blood tests [44]. As the results of this study show, most patients obtained the requested referral from their physician. Even physicians who took the time to thoroughly explain why the tests are unwarranted eventually gave the referral. Previous studies showed that doctors tend to give referrals even if they know these are unwarranted when they perceive patient expectations (23, 33). Most patients in our study shared they were willing to hear the physician’s view, but nonetheless expected their wish to be fulfilled. While in the past, medical information was the doctor’s domain, nowadays patients are exposed to a great deal of information on the internet. This has had a substantial effect on the physician-patient relationship [45, 46]. The internet offers a vast amount of information, much of which is not based on scientific studies [47]. Patients do not always know how to differentiate and choose reliable sources as a basis for their decision-making. Thus, despite the increasing exposure to information, the knowledge gap between physicians and their patients has, in fact, increased. This gap may lead to the public’s rising mistrust, with doctors perceived as representing the system’s interests rather than as advocates of the patient’s best interest [48].

The results also show that exposure to serious illness in someone close is an experience that continues to accompany the patient, instilling the belief that blood tests can prevent this from happening to them. Illness in the social environment was described before as a reason for blood test request [35]. It is interesting to note that in most cases in our results, patients did not request specific genetic tests or those recommended for screening in case of a genetic risk, but rather general blood tests, which they believe have the capacity to reveal any latent condition.

### Implications

The results of this study may have substantial practical implications. An in-depth understanding of the perceptions and views of healthy patients who request general blood tests may help develop interventions, both individual and community-based, educate patients about mistaken beliefs regarding the role of blood tests, promote patients’ health through healthy life-style maintenance and carrying out screening tests with proven efficacy, and minimize the use of tests that may be more harmful than beneficial. Furthermore, the study results may also add to our understanding of how doctors can deal more effectively with these requests while preserving and improving the patient-doctor relationship. Doctors should listen with empathy and try to understand patients’ fears and motives. They should tailor their explanations according to their acquaintance with the patient, sometimes taking a more scientific and factual approach while at other times addressing the uncertainty of life in accordance with the patient’s needs. In some circumstances doctors may choose to prescribe the tests to address and alleviate patients’ need to validate their health. The results of the current study can help doctors better understand just how widespread patients’ beliefs about the efficacy of blood tests really are. This understanding may alleviate doctors’ frustration when faced with requests for tests, thus fostering empathy and sensitivity towards their patients.

Furthermore, the study’s findings may help policy-makers deal with the issue of over-testing and the ensuing over-diagnosis and over-treatment, along with the costs these entail. Patient education programs may be developed to help patients understand the meaning of screening tests and the potential harms of superfluous tests. Doctors and health organizations should also be addressed. In addition, doctors should be educated about which screening tests are effective. Health organizations should make an extra effort to support doctors in dealing with requests for unnecessary tests that may harm both the individual patient and the medical system.

### Strengths and limitations

The current qualitative study focused on asymptomatic patients in order to pinpoint reasons for requesting unwarranted blood tests. This focus was also facilitated by the study population sample—patients who visited their doctor for the purpose of requesting blood tests. Interviews were conducted within two weeks of the visit, which allowed for a more detailed and in-depth investigation of participants’ perceptions and perspectives and added more information to previous findings regarding the reasons for requesting blood tests. The current study brings to our attention two important themes that, to the best of our knowledge, were not described before as reasons for patient requests for blood tests. These are the sense of responsibility to maintain one’s health and the need to monitor inner body functions.

There are a few limitations to this study. The sample size is quite small. Further studies among a wider population are warranted to shed more light on the matter. Quantitative studies may provide additional data, including the effect of the doctor’s explanation on the patient’s view. In addition, patients’ attitudes regarding routine blood tests are influenced by cultural and social trends, which vary in different countries and probably also in different areas in Israel. Additionally, the fact that patients who were sampled had recently requested screening tests means that they were likely to have more faith in screening tests than the general population. The patients′ experience after taking the blood tests was not directly addressed in the interview, as we focused mainly on the reasons for the request itself. Future research is needed to explore this aspect.

## Conclusions

This study shows that healthy asymptomatic patients request blood tests out of a sense of responsibility to their own health, a need for external validation of the body’s inner workings, and anxiety due to exposure to illness in a close acquaintance. Our results reflect a substantial gap between patients’ attitudes and beliefs about general blood tests and the current evidence-based guidelines. In order to minimize this gap, physicians need a better understanding of patients’ motivations so they can communicate more effectively with their patients on this topic. In addition, patient education programs can give patients more reliable information regarding screening tests, thus helping minimize the harm and costs of unnecessary tests.
